# Identification of Early Knee Osteoarthritis Based on Knee Joint Trajectory during Stair Climbing

**DOI:** 10.3390/ijerph192215023

**Published:** 2022-11-15

**Authors:** Ami Ogawa, Hirotaka Iijima, Masaki Takahashi

**Affiliations:** 1 Department of System Design Engineering, Faculty of Science and Technology, Keio University, Yokohama 223-8522, Japan; 2 Institute for Advanced Research, Nagoya University, Nagoya 464-8601, Japan; 3 Biomedical and Health Informatics Unit, Graduate School of Medicine, Nagoya University, Nagoya 461-8673, Japan

**Keywords:** IR-Locomotion, non-contact markerless measurement system, knee joint trajectory, early knee osteoarthritis, stair climbing

## Abstract

Patients with knee osteoarthritis show low stair climbing ability, but a diagnosis of stair performance time is not enough to identify the early stages of knee osteoarthritis. Therefore, we developed an indicator named range of the knee joint trajectory (RKJT) as a kinematic parameter to express more detailed characteristics than stair performance time. To achieve this, we used our developed “IR-Locomotion”, a markerless measurement system that can track the knee joint trajectory when climbing stairs. This study aimed to test whether the RKJT effectively identifies patients with early knee osteoarthritis even after controlling stair performance time. Forty-seven adults with moderate to severe knee pain (mean age 59.2 years; 68.1% women) underwent the radiographic examination (Kellgren and Lawrence grade) of both knees and a stair climbing test on 11 stairs. The RKJT during the stair climbing test was calculated by “IR-Locomotion”. A generalized linear mixed model was used to evaluate the discriminative capability of RKJT on early knee osteoarthritis (i.e., Kellgren and Lawrence grade of 1). As expected, patients with early knee osteoarthritis showed larger RKJT than non-radiographic controls (95% confidence interval: 1.007, 1.076). Notably, this finding was consistent even after adjusting stair performance time.

## 1. Introduction

Knee osteoarthritis (KOA) is one of the most common arthropathies in the elderly, causing pain and limited range of motion in the knee joint [1], eventually bringing total knee arthroplasty. There has been increasing interest in identifying the early stages of KOA, as no effective disease modifying treatments are available to postpone or prevent KOA. Early diagnosis improves treatment outcomes for patients with KOA [2,3].

Previous researchers have investigated the diagnostic ability of knee joint moments for early KOA during level walking, but these variables were unable to effectively identify early KOA [4,5,6]. Poor diagnostic ability may be due, at least in part, to level walking that is biomechanically easy for patients with early KOA. Biomechanically and physiologically, more challenging tasks may be suitable for identifying early KOA. Stair walking is known as one of the most demanding tasks in daily activities [7]. For example, the knee joint load during stair walking is greater than that during level walking [8,9,10]. Our previous study showed that patients with early KOA take longer to climb stairs [11]. This evidence suggests that stair climbing performance time may identify early KOA, but the prediction accuracy is inadequate [11]. In addition to stair climbing performance time, other parameters are required to effectively identify early KOA. Biomechanical outcomes such as joint movement are effective not only in diagnosing but also in understanding the mechanism of symptom progression. However, there is little research which is related to the early KOA [12].

An early KOA detection system is assumed to be installed in houses to daily diagnose the activities of people because few people will bother to seek medical attention in the unconscious early stages of the disease. As measuring activities at the house is delicate, an infrared-based depth sensor, which is non-contact and markerless, to measure kinematic parameters is reasonable at the point of considering privacy protection and body constraint-free. Our previously proposed measurement system, “IR-Locomotion” [13] acquires body joint trajectory based on a depth sensor. In this study, we used a modified version of “IR-Locomotion” [14] to reveal the kinematic characteristics of early KOA patients during stair climbing using for feasibility.

Knee flexion angle in the sagittal plane is known as a significant parameter that reflects the differences between KOA patients and controls [15]. However, the calculation of knee flexion angle using hip, knee, and ankle joint position has a risk of significant error because the depth data around ankle joint position has often noises as feet are always near stairs and are difficult to extract by the depth sensor. Thus, we hypothesized that the knee joint trajectory in the sagittal plane, which is more simply acquired and less affected by body shapes than knee flexion angle, reflects the characteristics of knee movements of early KOA patients. Since patients with KOA are likely to have less knee flexion [15] and lower external knee flexion moment when climbing stairs [16], the knee joint trajectory of early KOA patients is likely different from the controls. When the knee flexion angle, which mainly occurs in the sagittal plane, is slight, the displacement of the knee joint trajectory should be significant because the hip joint must be flexed significantly to keep the leg at a sufficient distance from the nosing line.

Our main aim in this paper was to explore the differences between early KOA patients and control groups based on knee joint trajectory, which can be acquired by “IR-Locomotion,” for the development of a system that can be introduced into daily life to screen for early KOA progression as a further aim. In terms of the Kellgren and Lawrence (K&L) grade, which indicates the level of KOA symptoms, the people suspected as early KOA patients with osteophytes or subchondral osteosclerosis are classified into K&L grade of 1, and people without such suspicion are classified into K&L grade of 0. A longitudinal study has shown that patients who are diagnosed with a K&L grade of 1 are more likely to worsen to a K&L grade of 2 or higher, which means that patients with a K&L grade of 1 are in the advanced stages of KOA. The importance of diagnosing KOA at an earlier stage is evident. Throughout the observation of the knee joint trajectories during stair climbing using “IR-Locomotion”, we hypothesized that early KOA patients perform a more significant knee joint trajectory than non-radiographic controls in the sagittal plane even after controlling the stair climbing time. This study aimed to examine the hypothesis of identifying early KOA patients using a knee joint trajectories-based indicator during stair climbing.

## 2. Materials and Methods

### 2.1. Participants

This study was a secondary cross-sectional baseline analysis of data from a randomized controlled trial [17]. The data of the community-based adults over the age of 50 interested in the measurement were collected via the internet. The inclusion criteria were (1) adults over the age of 50 years; (2) K&L values below grades 0 and 1 were assessed using weight-bearing anteroposterior radiographs for one or both knees; (3) Average pain experienced on a numerical rating scale was greater than three and less than ten over the previous month. The details were based on the previous report [17]. The targets of this study were distinguished into the K&L grade 0 group (control: OA identified without radiography) and the K&L grade 1 group (early KOA), focusing on early KOA detection. In this study, we did not check the type of KOA, so both primary and secondary KOA were mixed.

### 2.2. Radiographic Severity of Osteoarthritis

A trained examiner (HI) used the original version of the K&L grading system [18] to assess the radiographic severity of the tibiofemoral joints in both knees. Examiner had excellent credibility scores as both intra-examiner (κ: 0.876; 95% CI: 0.829, 0.924) and inter-examiner (κ: 0.845; 95% CI: 0.793, 0.897) [19].

### 2.3. Instrumentation of Stair Climbing Test

**Figure 1:**
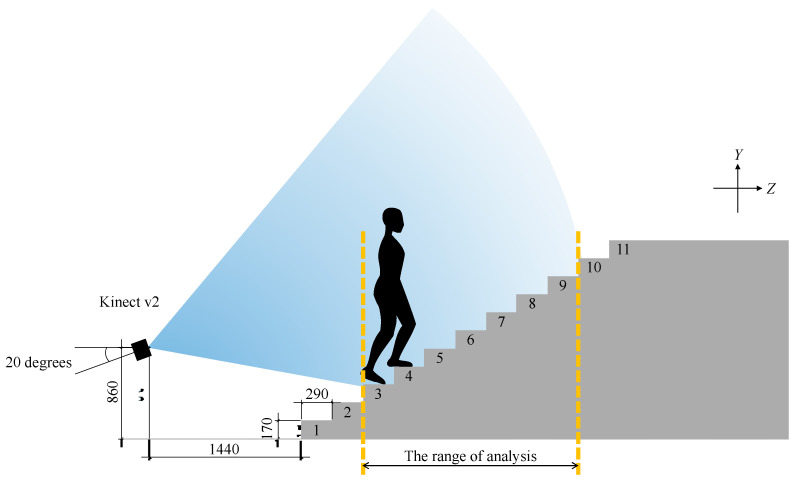
Experimental setup for stair climbing measurements using “IR-Locomotion” as a non-contact markerless system. We measured stairs climbing on an 11 steps staircase. The run length, riser height, and width were 290 mm, 170 mm, and 1350 mm, respectively. Kinect v2, a markerless RGB-D sensor, was set 1440 mm from the stairs at a height of 860 mm and 20 degrees tilted. Since the start and end of walking included acceleration and deceleration, namely a non-steady state, the knee joint trajectory of one step was extracted and analyzed from seven steps, excluding the first and the last two steps.

Kinect v2 (Microsoft, Redmond, WA, USA), an RGB-D sensor, was used to measure knee joint positions when climbing stairs. Kinect v2 has a skeleton tracking function that can automatically detect the position of joints based on the machine learning concept. However, it is often impossible to detect the knee joint positions when climbing stairs. The reason is that Kinect v2 does not contain the data acquired by the tilted sensor and needs to be tilted to capture the stair climbing [20]. Therefore, in this study, we used our previously suggested method to obtain the knee joint trajectories with Kinect v2 [13,14]. According to the previous method, we only used depth data captured from behind when climbing stairs. All data were sampled at 20 Hz. The experimental setup is shown in Figure 1. Kinect v2 was set at a distance of 1440 mm from the stairs, a height of 860 mm, and a tilt of 20 degrees as close as possible without interfering with walking so that it would fit within the angle of view. Since the start and end of walking included acceleration and deceleration (i.e., non-steady), the analysis range was set from an arbitrary step to the step before and was extracted from seven steps except the first and the last two steps.

### 2.4. Procedure for Eleven-Step Stair Climb Test (11-SCT)

Participants climbed the stairs wearing their clothes with specified typical shoes (LD AROUND M, Mizuno, Tokyo, Japan) as fast as possible and following the method recommended by OARSI [21]. In our 11-SCT, all participants started moving down the stairs due to environmental constraints, while participants normally moved up the stairs in the standard SCT. The 11-SCT contained two trials. The stairs consisted of 11 steps. The run length, riser height, and width of the stairway were 290 mm, 170 mm, and 1350 mm, respectively. Detailed information is provided in the previous report [17].

### 2.5. Range of Knee Joint Trajectory (RKJT)

**Figure 2:**
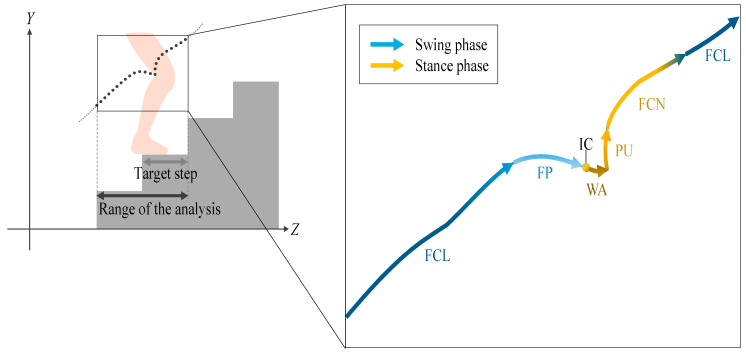
Representative image of knee joint trajectory in one cycle of stair climbing. One cycle of the knee joint trajectory was extracted in the range of two steps. The target step includes the stance phase and the step before it. The swing phase consisted of foot clearance (FCL) and foot placement (FP), while the stance phase started from the initial contact (IC) and consisted of weight acceptance (WA), pull-up (PU), and forward continuation (FCN).

Since there was no significant difference in the time of the 11-SCT between ascending and descending in both KOA and the control group [11], we focused on stair ascent in this paper. As an example, as shown in Figure 2, an arbitrary step knee joint trajectory in the sagittal plane was extracted for analysis. During stair walking, spatial position information of the body must be processed based on different reference planes between each step. The range of target steps on the depth (*Z*) axis was defined as the observed foot contact step, that is, the stair nosing of the step and the next step. One cycle of the knee joint trajectory was extracted for analysis in the range of two steps: the target step, including the stance phase, and the step before it. The stair climbing motion was divided into five sections: two for the swing phase and three for the stance phase, according to the previous method [22]. The swing phase consisted of foot clearance (FCL) and foot placement (FP), while the stance phase started from the initial contact (IC) and consisted of weight acceptance (WA), pull-up (PU), and forward continuation (FCN).

Since RKJT may change by the resolution of the analytical data depending on the distance from the sensor, the characteristics of the subject’s gait, and the step positions, it was confirmed in advance that there was no significant difference in the selection of analysis steps between groups. The arbitrarily extracted step positions for analysis did not follow a normal distribution pattern (Shapiro–Wilk test; *p*-value < 0.05), and the medians were the same at 7 (Mann–Whitney U-test; *p*-value = 0.601). 

**Figure 3:**
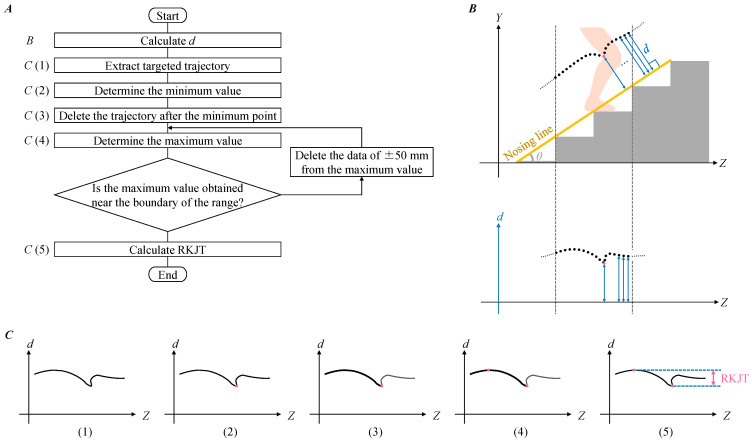
Definition and calculation procedure of range of knee joint trajectory (RKJT). (**A**) The flow of the procedure. (**B**) The distance (*d*) was calculated from the nosing line of the staircase to the knee joint trajectory. The black dotted line represents the knee joint trajectory. The nosing line of the staircase connects the stair nosing of all steps shown by the yellow line. The stair angle (*θ*) is the angle formed by the nosing line of the staircase and the horizontal lines (*Z*). (**C**) The description of the procedure is as follows: (1) The value *d* was calculated and extracted in the analytical range; (2) The minimum value was determined (pink dots); (3) The knee joint trajectory before the minimum value was extracted (bold black lines); (4) The maximum value was determined (pink dots); and (5) RKJT was calculated. When the maximum value was obtained near the boundary of the analysis range, the data of ±50 mm was deleted from the maximum value, and phase (4) was repeated.

We proposed a method to analyze the knee joint trajectories using our previous method. Figure 3 shows the procedure of the proposed method. The flow is shown in Figure 3A. First, we calculated the distance from each knee joint position to the nosing line that was lined between stair nosing and defined as *d* (Figure 3B). Next, the *d* value was extracted in the analysis range on the *Z* axis (Figure 3(C1)). The minimum value was only detected when the foot stepped on (Figure 3(C2)), while two maximum values were detected before and after the minimum value. Since this study focused on the ending of FCL to the beginning of PU, where the knee joint moves greater in the *d* direction, the maximum value occurred during the swing phase before the minimum value was adopted (Figure 3(C3,4)). Finally, the range of knee joint trajectory (RKJT), as the proposed index, was calculated as the difference between the maximum and minimum values of *d* (Figure 3(C5)). When people climbed by stepping on the edge of the steps, the maximum value deviated from the target step, so the range of analysis should include the entire cycle. On the other hand, the wrong maximum value could be adopted in another case because the range of analysis was too wide. Therefore, when the maximum value was obtained near the boundary of the range, the data of ±50 mm from the maximum value was deleted, and the maximum value was obtained again. The threshold was determined in a try-and-error manner. All processes were automated.

Of the two trial data, this study used only the second trial data. Case 1 (ICC (1, 2)) of the intraclass correlation coefficient (ICC) was applied to the RKJT data of both trials and assessed in duplicate. The result was 0.759 with good duplicability [23].

### 2.6. Patients Characteristics and Covariates

Patients self-reported their age, gender, and height. Weight was digitally measured with clothes on and shoes off. Body mass index (BMI) was calculated by dividing weight (kg) by height (m) squared. Knee pain severity and disability levels were evaluated by the Japanese Knee Osteoarthritis Measure (JKOM) subcategories of “pain and stiffness” (8 questions, 0–32 points) and “activities of daily living” (10 questions, 0–40 points) [24]. JKOM reflects the Japanese social and cultural background to globally standardized indexes. Comparisons with the Western Ontario and McMaster Universities Arthritis Index (WOMAC) [25] and the Medical Outcomes Study 36-Item Short-Form Health Survey (SF-36) [26] have shown adequate reliability and validity [24]. Knee pain during the 11-SCT trial was reported using a visual analog scale (VAS).

### 2.7. Statistical Analyses

Participants diagnosed with K&L grades of 1 and 0 were selected as the early KOA group and the control group (without radiographically identified KOA), respectively. RKJT were calculated based on their knee joint trajectories. Both left and right limb data from the second trial data were analyzed in this study. Differences in the RKJT mean values of each group were calculated by an appropriate test after performing the normality test and the equivariant test. The results of the Shapiro–Wilk’s test rejected the normality hypothesis (*p*-value = 0.019), and the results of Levene’s equal variance test adopted the equal variance hypothesis (*p*-value = 0.106). Therefore, the Mann–Whitney U-test was used to estimate differences between the mean values.

A generalized linear mixed model (GLMM) was used, and a binomial logistic regression analysis was performed to calculate the odds ratios and their 95% confidence intervals (CI) for the evaluation of the proposed index (continuous). Proposed index, limb side information (0: right; 1: left), age (continuous), gender, BMI (continuous), and VAS pain score during 11-SCT (continuous) were entered as independent variables. The K&L grade (0: K&L grade 0, 1: K&L grade 1) was entered as a dependent variable. Limb side information explained random effects on participant-specific parameters [27,28]. The remaining independent variables explained fixed effects. Age, gender, BMI, and VAS pain scores were entered as covariates. All statistical analyses were performed using IBM SPSS Statistics, version 25 (IBM, Armonk, NY, USA). The *p*-values less than 0.05 were considered significant.

## 3. Results

Fifty-nine participants were included in the test. Eight participants were excluded because of wearing oversized pants, which could reduce the accuracy of the data. Both knees were individually considered. This means that 102 samples were calculated from the data of 51 participants. Among them, 90 samples with K&L grade 0 or 1 were analyzed. In addition, due to systematic measurement issues and clothing issues, the data of the first trial were alternatively used in nine samples. According to Table 1, only K&L grade 1 participants were analyzed as early KOA patients (*n* = 20 participants, *n* = 35 knees), and K&L grade 0 participants were taken as controls into account (*n* = 27 participants, *n* = 55 knees). In four participants, only one knee was analyzed because the K&L grade of the other knee exceeded 1. There were three controls and one early KOA.

**Figure 4:**
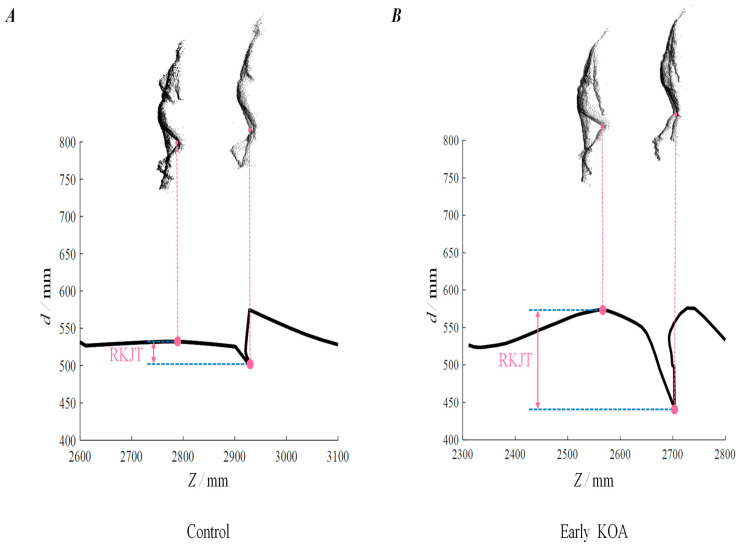
Comparison of RKJT values for K&L grades 0 and 1. (**A**) shows data for participants with K&L grade 0, and (**B**) shows data for K&L grade 1. The black lines show the analysis range, and the pink dots show the maximum and minimum points extracted. The four silhouettes correspond to the maximum and minimum values.

Figure 4 shows a visual comparison of the law knee joint trajectory between controls (Figure 4A) and early KOA (Figure 4B) patients with similar height and BMI. 

Table 2 compares the RKJT differences between people with and without early KOA. Early KOA people showed that RKJT was 10 mm larger than control group people (95% CI: −17.6, −2.39 mm; *p*-value = 0.038). Binomial logistic regression shows that a 1 mm increase in the proposed index was significantly associated with a 1.04-fold increase in the odds ratio of early KOA after adjusting for covariates. Early KOA people seem to perform 11-SCT [11] for longer. Therefore, when we considered the stopwatch-based 11-SCT time as an additional covariate, we confirmed whether the identified relationship between RKJT and early KOA was similar. In fact, after adding 11-SCT in covariates (odds ratio: 1.037; 95% CI: 1.001, 1.073; *p*-value = 0.044), we found that the early KOA displayed a significantly larger RKJT.

In addition, to infer the function of RKJT in early KOA, we calculated the RKJT correlation between the maximum and minimum values. The correlation between RKJT and the minimum value (Pearson’s correlation coefficients: −0.405; 95% CI: −0.624, −0.126; *p*-value = 0.006) was shown to be higher than the correlation between RKJT and the maximum value (Pearson’s correlation coefficients: 0.296; 95% CI: 0.002, 0.542; *p*-value = 0.049).

The results without the knees which another knee was diagnosed as having more than a K&L grade of 1, showed the same tendency. The same tendency was also shown in the results, including a K&L grade of 2 in the early KOA group.

## 4. Discussion

This study hypothesized that RKJT in early KOA was greater than the controls during steady stair climbing. Ninety samples of knee joint trajectories with K&L grades of 0 and 1 were analyzed. The average RKJT in early KOA patients was significantly greater than that in controls, supporting the hypothesis. The results showed that RKJT could identify early KOA patients.

### 4.1. Interpretation of Larger RKJT in People at Early KOA

Patients at early KOA showed larger RKJT than the control group, which means larger knee joint movement in the sagittal plane. This indicates two theories: (1) Early KOA patients bend their lower thighs forward and step deeply in the stance phase. (2) They raise their lower limbs high during the swing phase. The first theory leads to a smaller minimum value of knee joint trajectory, and the second theory leads to a larger maximum value. The correlation coefficient results indicate that the correlation between RKJT and the minimum value was greater than the correlation between RKJT and the maximum value, supporting the first theory. It can be considered that early KOA patients unintentionally adopted a strategy that puts a high knee load on WA. 

The larger RKJT in early KOA was independent of these factors, as GLMM included age, gender, height, BMI, and VAS pain scores. Notably, this trend was similar after the addition of 11-SCT time, indicating that poor stair walking performance cannot explain the mechanism of larger RKJT in early KOA.

### 4.2. Significance and Clinical Impacts of Study

The development of a screening index in early KOA has not been achieved in previous studies [4,5,6]. In this study, a new finding was that the range of the knee trajectories during stair climbing, perpendicular to the nosing line, was significantly larger in patients with early KOA. The results showed the importance of observing the knee joint trajectories in early KOA screening. In addition, we proposed a simple diagnostic system that enables frequent diagnosis. Kinetic parameters such as knee contact forces and external moments have been considered in previous studies [6], but it was challenging to set up a device to record kinetic parameters, such as force plates that must be embedded at each step. The findings are clinically valuable because a simple measurement system can identify patients at high risk of KOA, either at home or in rehab centers. Identifying patients with early KOA could be a good screening tool for inclusion in clinical trials aiming to postpone its radiological progression.

### 4.3. Study Limitations and Strengths

It is not easy to interpret the difference between RKJT with these results biomechanically. However, the results show the ability of RKJT to distinguish people with K&L grades of 0 and 1.

As the control group without radiographic KOA markers also complained of knee pain, the results may not be applicable to asymptomatic healthy subjects. However, pain cannot explain the difference between K&L grades 0 and 1 because the VAS pain score was added to the covariates. Additionally, this study could not consider the effect of patellar-femoral joint arthrosis as a confounding factor as we did not have skyline merchant views. Still, at least a part of the effect of patellar-femoral joint arthrosis is considered by adding the VAS pain score related to patellar-femoral joint arthrosis to the covariates, and the results were robust.

The participants may be unable to walk normally because of wearing specified shoes during the test. Instead, the effect of shoes was eliminated by unifying the shoe conditions to reveal the feasibility of our system. On the other hand, we cannot conclude that the effects of the users’ clothes or the physical conditions, which we did not unify the requirements, are negligible only from this study.

“IR-Locomotion” has no installation constraints because there is no need to use large equipment or attach devices to the subject. RKJT has strength in its simplicity for acquisition compared with other kinematic and kinetic parameters. Therefore, early diagnosis of KOA using RKJT may be possible in daily life. This study only validated the use of a diagnostic system on one type of staircase. However, walking also depends on the slope of the stairs. Future work will validate staircase designs that match those of the house.

## 5. Conclusions

To distinguish between early KOA (K&L grade 1) and control (K&L grade 0) patients, we proposed a new index RKJT that uses the knee joint trajectory when climbing stairs. The results showed a significant relationship between RKJT and K&L grades. Furthermore, RKJT was significantly correlated with K&L grade classification independent of 11-SCT time. These findings provide mechanistic insights into early KOA-related changes in biomechanics.

## Figures and Tables

**Table 1:** Characteristics of participants and their knees.

Person-Level Characteristics	All (*n* = 47 Participants)	Control (*n* = 27 Participants)	Early KOA (*n* = 20 Participants)
Age, years	59.2 ± 5.98	58.7 ± 6.18	60.0 ± 5.79
Female, no. (%)	32 (68.1)	17 (63.0)	15 (75.0)
Height, m	1.61 ± 0.0817	1.62 ± 0.0853	1.61 ± 0.0782
Mass, kg	59.2 ± 10.1	57.3 ± 8.75	61.8 ± 11.5
BMI, kg/m^2^	22.7 ± 3.04	21.8 ± 2.61	23.9 ± 3.26
Bilateral disease, no. (%) †	16 (34.0)	0 (0.0)	16 (80.0)
VAS pain score during 11-SCT, mm	14.3 ± 16.5; 8 [0, 63] *	10.7 ± 11.3; 4 [0, 34] *	19.2 ± 21.0; 10 [0, 63] *
JKOM, points			
Pain and stiffness	6.70 ± 4.14; 6 [0, 22] *	5.59 ± 3.48; 5 [1, 16] *	8.20 ± 4.56; 7 [0, 22] *
Activities of daily living	2.89 ± 3.30; 2 [0, 14] *	2.00 ± 2.47; 1 [0, 8] *	4.10 ± 3.92; 3 [0, 14] *
Participation in social activities	2.66 ± 2.05; 2 [0, 9] *	2.15 ± 1.68; 2 [0, 7] *	3.35 ± 2.32; 3 [0, 9] *
General health conditions	1.96 ± 1.02; 2 [0, 4] *	1.78 ± 1.05; 2 [0, 3] *	2.20 ± 0.95; 2 [0, 4] *
Total score	14.2 ± 8.22; 12 [3, 49] *	11.5 ± 5.79; 10 [3, 25] *	17.9 ± 9.68; 16 [6, 49] *
Knee-level characteristics	All (*n* = 90 knees)	Control (*n* = 55 knees)	Early KOA (*n* = 35 knees)
K&L grade, no. (%)			
Grade 0	55 (61.1)	55 (100.0)	0 (0.0)
Grade 1	35 (38.9)	0 (0.0)	35 (100.0)

BMI, body mass index; JKOM, Japanese Knee Osteoarthritis Measure; K&L grade, Kellgren and Lawrence grade; KOA, knee osteoarthritis; VAS, visual analog scale; 11-SCT, 11-step stair climb test. Unless otherwise stated, the values are mean ± SD. * Median [lower range–upper range] was provided because of the scattered distribution of the answered items. † Bilateral disease was defined as K&L grade ≥1 in both knees. Participants diagnosed with at least one K&L grade 1 knee were considered early KOA patients.

**Table 2:** Results of a binary logistic regression analysis investigating the relationship between RKJT and the presence of early KOA.

Independent Variable	Control (*n* = 55 Knees)	Early KOA (*n* = 35 Knees)	Difference between Control and Early KOA	Model 1 † Control vs. Early KOA		Model 2 †† Control vs. Early KOA	
	Mean ± SD	Mean ± SD	Mean (95% CI)	OR (95% CI)	*p*-Value	OR (95% CI)	*p*-Value
RKJT, mm	61.7 ± 18.4	71.7 ± 16.5	**10.0** **(−17.6, −2.39)**	**1.04** **(1.01, 1.08)**	**0.018**	**1.04** **(1.00, 1.07)**	**0.044**

RKJT, range of knee joint trajectory; KOA, knee osteoarthritis; OR, odds ratio; 95% CI, confidence interval. † For the presence of early KOA, Model 1 was calculated to indicate the predictive ability of independent variables and simultaneously included (GLMM) age (continuous), female gender, body mass index (continuous), and VAS pain score during 11-SCT (continuous) in the binary logistic regression model. †† For the presence of early KOA, Model 2 was calculated to indicate the predictive ability of independent variables and simultaneously included (GLMM) and the same variables as model 1 and the stopwatch-based 11-SCT time in the binary logistic regression model. Bold fonts represent statistically significant results.

## Data Availability

Not applicable.

## References

[1] Guccione A.A., Felson D.T., Anderson J.J., Anthony J.M., Zhang Y., Wilson P.W., Kelly-Hayes M., A Wolf P., E Kreger B., Kannel W.B. (1994). The effects of specific medical conditions on the functional limitations of elders in the Framingham Study. Am. J. Public Health.

[2] Guermazi A., Niu J., Hayashi D., Roemer F.W., Englund M., Neogi T., Aliabadi P., McLennan C.E., Felson D. (2012). Prevalence of abnormalities in knees detected by MRI in adults without knee osteoarthritis: Population based observational study (Framingham Osteoarthritis Study). BMJ.

[3] Bruyere O., Cooper C., Arden N., Branco J., Brandi M.L., Herrero-Beaumont G., Berenbaum F., Dennison E., Devogelaer J.-P., Hochberg M. (2015). Can we identify patients with high risk of osteoarthritis progression who will respond to treatment? A focus on epidemiology and phenotype of osteoarthritis. Drugs Aging.

[4] Duffell L.D., Southgate D.F., Gulati V., McGregor A.H. (2014). Balance and gait adaptations in patients with early knee osteoarthritis. Gait Posture.

[5] Mahmoudian A., van Dieёn J.H., Baert I.A., Bruijn S.M., Faber G.S., Luyten F.P., Verschueren S.M. (2017). Changes in gait characteristics of women with early and established medial knee osteoarthritis: Results from a 2-years longitudinal study. Clin. Biomech..

[6] Meireles S., de Groote F., Reeves N., Verschueren S., Maganaris C., Luyten F., Jonkers I. (2016). Knee contact forces are not altered in early knee osteoarthritis. Gait Posture.

[7] Larsen A.H., Puggaard L., Hämäläinen U., Aagaard P. (2008). Comparison of ground reaction forces and antagonist muscle coactivation during stair walking with ageing. J. Electromyogr. Kinesiol..

[8] Bergmann G., Deuretzbacher G., Heller M., Graichen F., Rohlmann A., Strauss J., Duda G. (2001). Hip contact forces and gait patterns from routine activities. J. Biomech..

[9] Kutzner I., Heinlein B., Graichen F., Bender A., Rohlmann A., Halder A., Beier A., Bergmann G. (2010). Loading of the knee joint during activities of daily living measured in vivo in five subjects. J. Biomech..

[10] Liikavainio T., Isolehto J., Helminen H.J., Perttunen J., Lepola V., Kiviranta I., Arokoski J.P., Komi P.V. (2007). Loading and gait symmetry during level and stair walking in asymptomatic subjects with knee osteoarthritis: Importance of quadriceps femoris in reducing impact force during heel strike?. Knee.

[11] Iijima H., Eguchi R., Shimoura K., Aoyama T., Takahashi M. (2019). Stair climbing ability in patients with early knee osteoarthritis: Defining the clinical hallmarks of early disease. Gait Posture.

[12] Emery C.A., Whittaker J.L., Mahmoudian A., Lohmander L.S., Roos E.M., Bennell K.L., Toomey C.M., Reimer R.A., Thompson D., Ronsky J.L. (2019). Establishing outcome measures in early knee osteoarthritis. Nat. Rev. Rheumatol..

[13] Ogawa A., Mita A., Yorozu A., Takahashi M. (2017). Markerless knee joint position measurement using depth data during stair walking. Sensors.

[14] Ogawa A., Iijima H., Takahashi M. (2021). Staircase design for health monitoring in elderly people. J. Build. Eng..

[15] Hicks-Little C.A., Peindl R.D., Hubbard T.J., Scannell B.P., Springer B.D., Odum S.M., Fehring T.K., Cordova M.L. (2011). Lower extremity joint kinematics during stair climbing in knee osteoarthritis. Med. Sci. Sport. Exerc..

[16] Iijima H., Shimoura K., Aoyama T., Takahashi M. (2018). Biomechanical characteristics of stair ambulation in patients with knee OA: A systematic review with meta-analysis toward a better definition of clinical hallmarks. Gait Posture.

[17] Shimoura K., Iijima H., Suzuki Y., Aoyama T. (2019). Immediate effects of transcutaneous electrical nerve stimulation on pain and physical performance in individuals with preradiographic knee osteoarthritis: A randomized controlled trial. Arch. Phys. Med. Rehabil..

[18] Kellgren J., Lawrence J. (1957). Radiological assessment of osteo-arthrosis. Ann. Rheum. Dis..

[19] Iijima H., Suzuki Y., Aoyama T., Takahashi M. (2018). Interaction between low back pain and knee pain contributes to disability level in individuals with knee osteoarthritis: A cross-sectional study. Osteoarthr. Cartil..

[20] Ogawa A., Mita A., Georgoulas C., Bock T. A Face Recognition System for Automated Door Opening with parallel Health Status Validation Using the Kinect v2, ISARC. Proceedings of the International Symposium on Automation and Robotics in Construction.

[21] Dobson F., Hinman R.S., Hall M., Terwee C., Roos E.M., Bennell K. (2012). Measurement properties of performance-based measures to assess physical function in hip and knee osteoarthritis: A systematic review. Osteoarthr. Cartil..

[22] McFadyen B.J., Winter D.A. (1988). An integrated biomechanical analysis of normal stair ascent and descent. J. Biomech..

[23] Koo T.K., Li M.Y. (2016). A guideline of selecting and reporting intraclass correlation coefficients for reliability research. J. Chiropr. Med..

[24] Akai M., Doi T., Fujino K., Iwaya T., Kurosawa H., Nasu T. (2005). An outcome measure for Japanese people with knee osteoarthritis. J. Rheumatol..

[25] Bellamy N., Buchanan W.W., Goldsmith C.H., Campbell J., Stitt L.W. (1988). Validation study of WOMAC: A health status instrument for measuring clinically important patient relevant outcomes to antirheumatic drug therapy in patients with osteoarthritis of the hip or knee. J. Rheumatol..

[26] McConnell S., Kolopack P., Davis A.M. (2001). The Western Ontario and McMaster Universities Osteoarthritis Index (WOMAC): A review of its utility and measurement properties. Arthritis Care Res. Off. J. Am. Coll. Rheumatol..

[27] Stewart S., Pearson J., Rome K., Dalbeth N., Vandal A.C. (2018). Analysis of data collected from right and left limbs: Accounting for dependence and improving statistical efficiency in musculoskeletal research. Gait Posture.

[28] Hannigan J., Chou L.-S. (2019). Sex differences in lower extremity coordinative variability during running. Gait Posture.

